# Genome Sequence of a Bornavirus Recovered from an African Garter Snake (*Elapsoidea loveridgei*)

**DOI:** 10.1128/genomeA.00779-14

**Published:** 2014-10-09

**Authors:** Mark D. Stenglein, Eli B. Leavitt, Michael A. Abramovitch, Jimmy A. McGuire, Joseph L. DeRisi

**Affiliations:** a Department of Biochemistry and Biophysics, University of California San Francisco, San Francisco, California, USA; b Department of Integrative Biology and Museum of Vertebrate Zoology, University of California, Berkeley, Berkeley, California, USA; c Howard Hughes Medical Institute, Chevy Chase, Maryland, USA

## Abstract

Bornaviruses are known to infect mammals and birds, and they have been associated with disease in both groups of animals. Here, we report the genome sequence of a bornavirus identified in a wild-caught Loveridge’s garter snake (*Elapsoidea loveridgei*).

## GENOME ANNOUNCEMENT

The field of reptile virology is significantly understudied relative to that of viruses that infect mammalian species. However, the field is developing quickly, thanks to growing interest and the advent of newer technologies for detecting novel viruses. Many representatives of well-studied viral families have been characterized in reptile species (1–3), and more recently, deep-sequencing approaches have identified entire new clades of viruses from families previously not known to infect reptiles (4, 5).

Bornaviruses (family *Bornaviridae*) are negative-strand RNA viruses that have been associated with disease in horses and, more recently, with proventricular dilatation disease (PDD) in parrots and other psittacines (6, 7). In 2010, fragments containing the X and phosphoprotein (P) genes of a putative bornavirus were recognized in a cDNA library prepared from Gaboon viper (*Bitis gabonica*) RNA (8, 9). To date, a complete genome of a reptile bornavirus (RBV) has yet to be elucidated.

Through a deep-sequencing survey of reptile samples housed in the Field Museum of Natural History, we discovered large numbers of sequences with similarity to those of avian bornaviruses originating from the tissues of an *Elapsoidea loveridgei* (Loveridge’s garter snake), specimen FMNH 251327. Initial short contigs were generated with Trinity (10), followed by iterative contig extension using the *de novo* assembler PRICE (11). The resulting assembly consisted of a single 8,884-nucleotide contig. The annotation of the sequence revealed a canonical gene repertoire (N, X, P, M, G, and L) and configuration for bornaviruses, including sequence motifs, such as transcription initiation sites, transcriptional terminators, and a splice donor site for the intron present in L (polymerase). The remapping of the individual sequencing reads revealed a mean coverage of 200-fold (±91).

A comparison of the nucleotide sequence to that of other bornaviruses revealed that this virus is related to the avian bornaviruses. For example, avian Borna disease virus (isolate bil, GenBank accession no. EU781967) shares 62% pairwise global nucleotide identity. Interestingly, the alignment of the putative P protein sequence with the previously reported RBV P is only 64% identical at the amino acid level, suggesting the existence of a large diverse family of bornaviruses present in reptiles, although no disease association has been recognized to date.

Based on alignment to avian Borna disease virus, we believe this assembly represents the complete genome (12) and thus the first bornavirus genome sequenced from a nonavian reptile.

### Nucleotide sequence accession number.

The genome sequence has been deposited in GenBank under accession no. KM114265.
